# Eltrombopag and its beneficial role in management of ulcerative Colitis associated with ITP as an upfront therapy case report

**DOI:** 10.1002/ccr3.3783

**Published:** 2021-01-21

**Authors:** Elabbass A. Abdelmahmuod, Elrazi Ali, Mohanad A. Ahmed, Mohamed A. Yassin

**Affiliations:** ^1^ Department of Internal Medicine Hamad Medical Corporation Doha Qatar; ^2^ Department of Hematology and Medical Oncology National Center for Cancer Care and Research Doha Qatar

**Keywords:** eltrombopag, IBD, inflammatory bowel diseases, ITP, ulcerative colitis

## Abstract

Eltrombopag can be used safely as upfront medication in the management of ulcerative colitis as well as ITP, and it showed a beneficial effect in both disorders.

## Graphical abstract

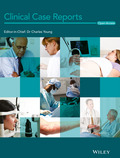

## 1 INTRODUCTION

Ulcerative colitis is an inflammatory bowel disease that affects the gastrointestinal tract characterized by abdominal pain, bloody diarrhea, malnutrition, malabsorption, and weight loss. It involves only the colon, and the commonest sides are the rectum and sigmoid colon. Immune thrombocytopenic purpura (ITP) is an autoimmune disorder characterized by autoantibodies directed against the platelet antigen leading to thrombocytopenia. However, the commonest extraintestinal manifestations of ulcerative colitis are arthritis, mouth ulcer, uveitis, and pyoderma gangrenosum. There are a few cases reported that showed there is an association between ulcerative colitis and ITP. Eltrombopag (thrombopoietin receptor agonist) use in the management of chronic ITP, its role in activation hematopoietic bone marrow receptors of the platelets. Management of ulcerative colitis is immunosuppressive medication; however, here we report A 22 year old who has ulcerative colitis and ITP received Eltrombopag and discovered that his endoscopy finding showed remission of ulcerative colitis.

Ulcerative colitis (UC) is an autoimmune inflammatory disorder characterized by an ulcer of the colon and rectum. The primary etiology is unknown; however, there are multiple factors that play a role in the pathogenesis of UC, such as genetic factors (HLA system), alteration of normal bowel flora, immune dysfunction, and environmental factors.^1^

Ulcerative colitis is classified based on the site of inflammation into six classes, which include proctitis, proctosigmoiditis, left side colitis, pancolitis or universal colitis, and fulminant colitis.^2^

An extraintestinal manifestation of ulcerative colitis includes spondylosis, skin involvement like pyoderma gangrenosum, primary sclerosing cholangitis, eye involvement (Anterior uveitis) systems. However, ITP is extremely rare associated with UC, but few cases showed these associations.^3^

Immune thrombocytopenia (ITP) is an autoimmune disorder characterized by autoantibodies directed against platelet surface antigens that lead to decreased platelet numbers and manifest as skin bleeding or manifested as systemic in severe cases bleeding like intracranial hemorrhage (ICH). ITP can be acquired or inherited disorders.^4^

Eltrombopag (EPAG) is a thrombopoietin agonist that can be used orally to enhance platelet production from the bone marrow. It is used for the treatment of chronic ITP, especially for those who are resistant to steroids.^5^

Standard treatment for ulcerative colitis depends on the extent of involvement and disease severity. UC can be treated with several medications, including 5‐ASA drugs such as sulfasalazine and mesalazine and corticosteroids such as prednisone^6^.

However, here we present the responsiveness of UC to EPAG that is used for the treatment of UC associated ITP.

## 2 CASE PRESENTATION

A 21‐year‐old gentleman with no chronic medical conditions has been suffering from diarrhea for the last 4‐6 months. He has 10‐12 bowel motions on average per day. The volume and consistency differ each time, but mostly it is watery (mucous) and moderate amount.

He also has on and off blood coming with stool, mild to moderate in amount. Sometimes, he also has abdominal cramps, which improve with the bowel motion. He denied nausea/vomiting. He also denied any fever, weight loss, loss of appetite. There is no joint pain and vision problems. He had 2‐3 Visits to ED and PHCC, and he was managed symptomatically with IBS and hemorrhoids.

**FIGURE 1:**
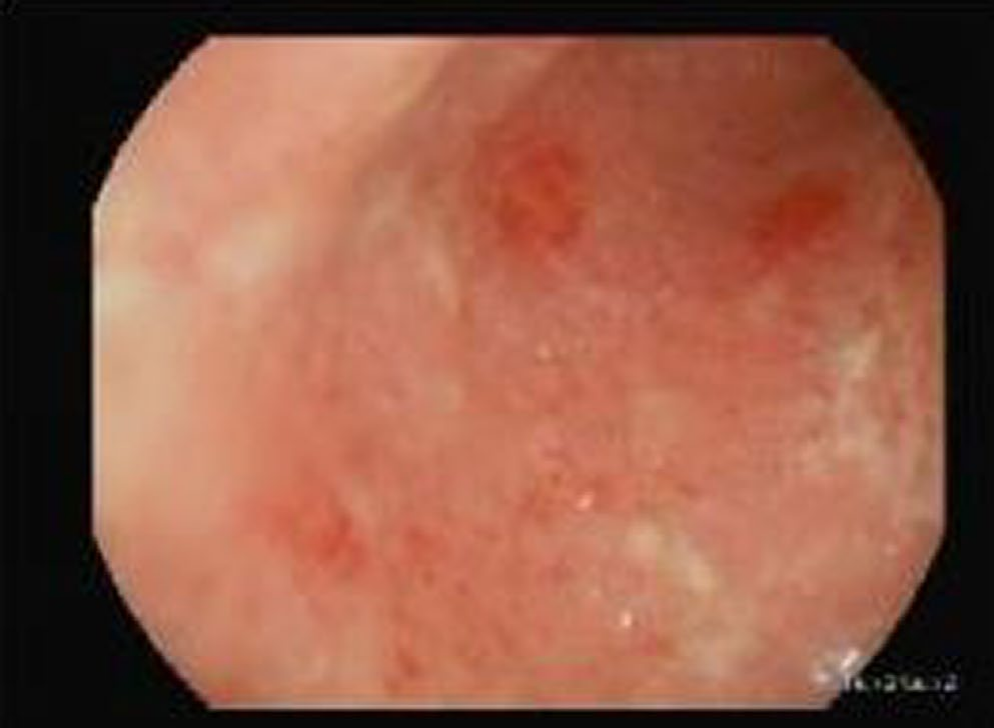
Showed marked erythema, mucosal friability of Sigmoid colon, reflect of sigmoid colitis

**FIGURE 2:**
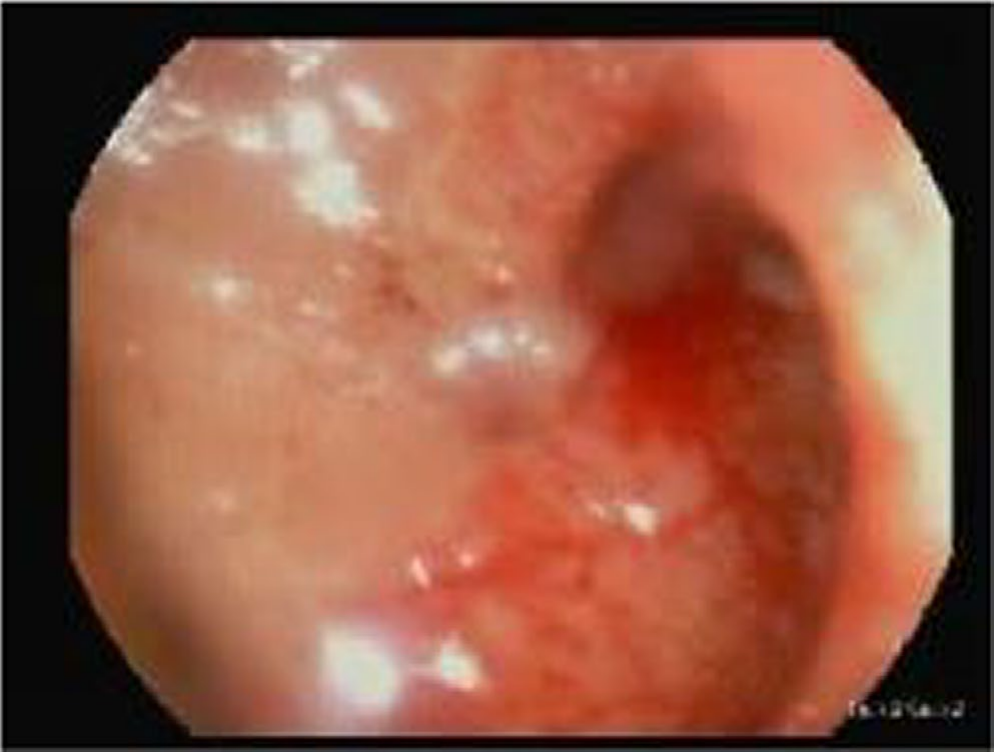
Showed erythema, mucosal friability and multiple ulceration of rectum, reflect proctitis

He underwent sigmoidoscopy and showed ulceration in the rectum and sigmoid colon, and features consistent with ulcerative colitis. (see Figures 1 and 2). A biopsy sent, and the result showed:

Left colon, biopsy showed.

Intramucosal lymphoid aggregates.

Rectum and sigmoid biopsies showed Chronic active colitis, moderate. Negative for granuloma, dysplasia, or carcinoma.

Full colon colonoscopy was done and showed proctosigmoiditis, mayo grade 2‐3, same findings of sigmoidoscopy.

**FIGURE 3:**
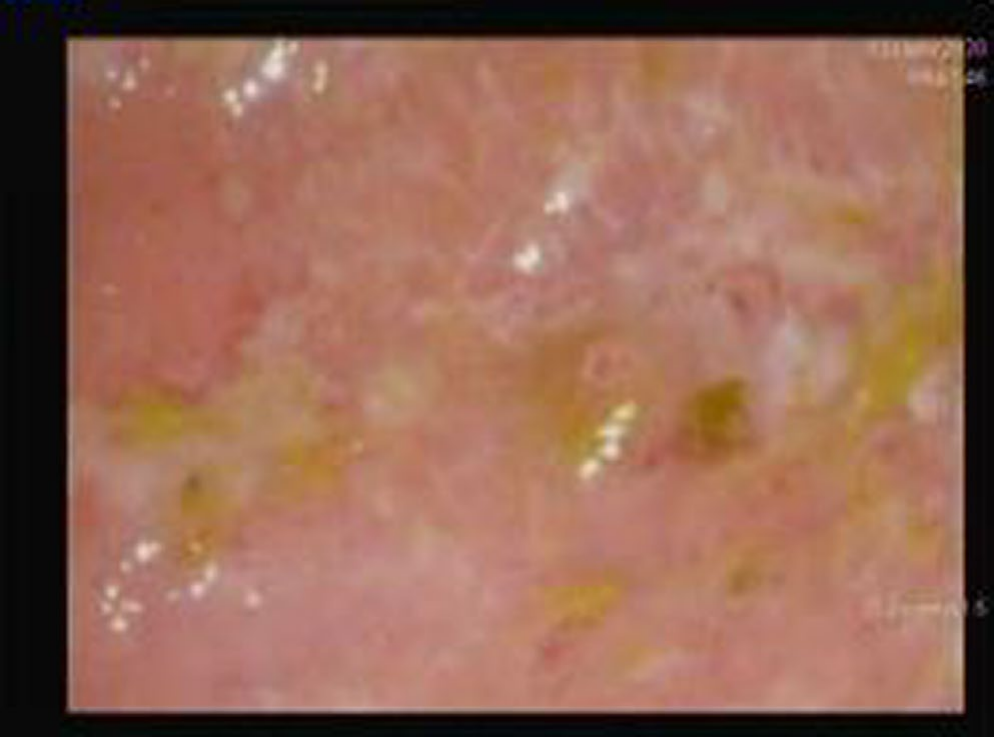
Showed marked reduction in erythema, mucosal friability and ulcer in both sigmoid colon and rectum, reflect remission of UC

**FIGURE 4:**
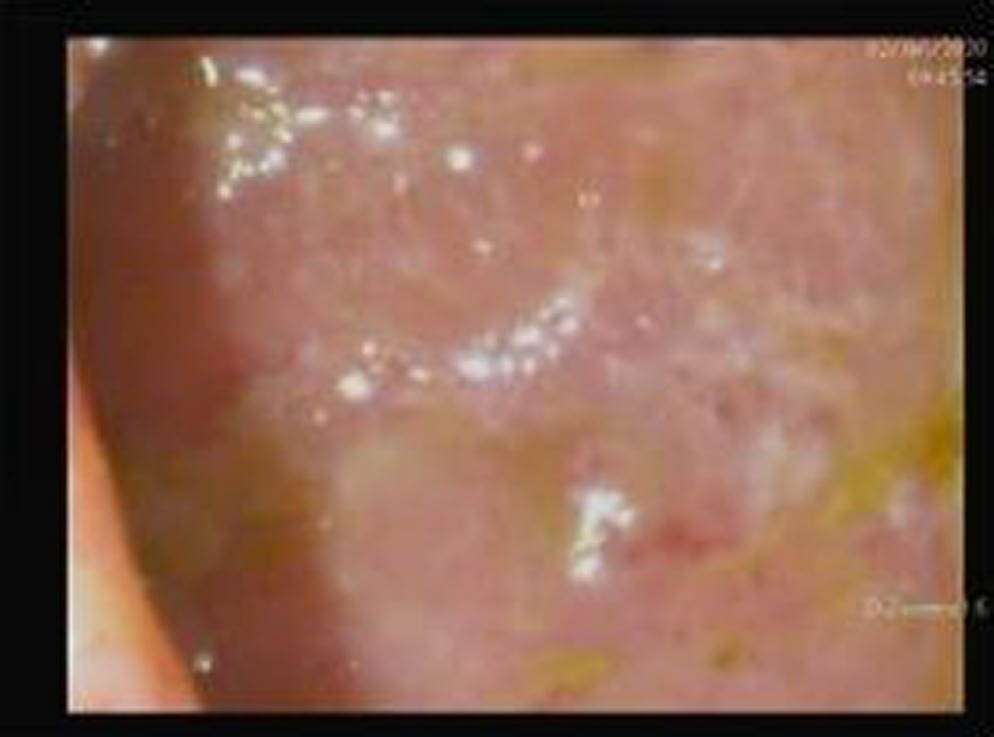
Showed marked reduction in erythema, mucosal friability and ulcer in both sigmoid colon and rectum, reflect remission of UC

His initial complete blood count showed thrombocytopenia and high calprotectin (see Table 1), which was labeled as ITP after being excluding the other causes of thrombocytopenia. He started on mesalamine topical and oral (Mesalazine tabs 400 mg BID for two months) and Eltrombopag (Dosage of Eltrombopag was 50 mg orally 4 times /day for 4 months) for ITP after he refused steroids, and he has been followed in the clinic for the response to the management. Repeated colonoscopy after eight weeks showed a significant decrease in sigmoidoproctitis (see Figures 3 and 4) and the improvement of his platelets (see Table 2).

**TABLE 1:** Low platelet and high Calprotectin

Detail	Value w/Units	Flags	Normal Range
Hemoglobin	13.7 gm/dL	normal	13.0‐17.0
Platelet	35 × 10^3/μL	low	150‐400
Calprotectin	1,108 mg/Kg	High	5‐50

**TABLE 2:** showed improved platelets

Details	Normal Range	Flags	Value w/Units
Platelets	163 x10^3/μL	150‐400	normal

## 3 DISCUSSION

Inflammatory bowel disease (ulcerative colitis and Crohn disease) both can be presented with intestinal manifestations such as bloody diarrhea, tenesmus, abdominal pain, malabsorption, and weight loss.^7^ However, inflammatory bowel diseases can be present with extraintestinal manifestation such as musculoskeletal (arthritis ), mouth (aphthous ulcer ), ocular ( uveitis), and hepatobiliary ( primary biliary cirrhosis and primary sclerosing cholangitis ). ITP is one of the rare extraintestinal manifestations of IBD.^8^

ITP is characterized by systemic hemorrhagic diathesis due to excessive thrombocyte destruction. ITP can be classified into acute (newly diagnosed) and chronic types according to clinical course. In general, acute ITP is more common in infants than adults and often recovers spontaneously, whereas chronic ITP is typically recurrent and occasionally refractory to therapy.^9^ ITP is a disease of exclusion, and it is diagnosed by low platelet enlargement of megakaryocytes in the bone marrow.^10^

Upon the literature review, there are a few cases that reported the association between IBD and ITP. However, there are no clear data support that ITP is common among Crohn's disease and ulcerative colitis.

There is no clear data provided to know the underlying pathogenesis of ITP related to IBD. However, some theories showed there is antigen similarity between platelet surface antigen and gut lumen, particularly bacterial surface antigen, in addition to the increasing level of TH1 and CD4 in both ITP and IBD.^11^

Standard management of UC is mesalamine, steroids, and immunosuppressive medications. However, when was started Eltrombopag for ITP, the sour patient showed marked improvement in both platelets and regression of UC.

Eltrombopag is a small‐molecule nonpeptide TPO‐R agonist administered orally. It can effectively increase platelet counts and reduce bleeding events in patients with chronic ITP, with an overall response rate of 60% to 80%. Eltrombopag is well tolerated and has a good safety profile in adults.^12^

Upon literature review, there are no studies or case reports that document the association between Eltrombopag and regression of IBD. Despite there is no clear pathogenesis, but the immunomodulatory effect of Eltrombopag could decrease the inflammatory response of IBD.

## 4 CONCLUSION

Eltrombopag can be used safely as upfront medication in the management of ulcerative colitis and ITP, and it showed a beneficial effect in both disorders.

## CONFLICT OF INTEREST

None declared.

## AUTHOR CONTRIBUTIONS

Elabbass Abdulmahmuod: writing editing, final approval. Elrazi Ali: writing editing, final approval. Mohamed Yassin: writing editing, final approval. Mohanad Ahmed: writing editing, final approval.

## STATEMENT OF ETHICS

This case was approved by the Hamad Medical Corporation's Medical Research Center, And the patient consented to the publication of his case.

## Data Availability

Data available on request.
